# Clearly Transparent Nanopaper from Highly Concentrated Cellulose Nanofiber Dispersion Using Dilution and Sonication

**DOI:** 10.3390/nano8020104

**Published:** 2018-02-12

**Authors:** Takaaki Kasuga, Noriyuki Isobe, Hitomi Yagyu, Hirotaka Koga, Masaya Nogi

**Affiliations:** 1 Graduate School of Engineering, Osaka University, Mihogaoka 8-1, Ibaraki, Osaka 567-0047, Japan; tkasuga@eco.sanken.osaka-u.ac.jp; 2 Research and Development Center for Marine Biosciences, Japan Agency for Marine-Earth Science and Technology (JAMSTEC), 2-15 Natsushima-cho, Yokosuka, Kanagawa 237-0061, Japan; isoben@jamstec.go.jp; 3 The Institute of Scientific and Industrial Research, Osaka University, Mihogaoka 8-1, Ibaraki, Osaka 567-0047, Japan; yagyu@eco.sanken.osaka-u.ac.jp (H.Y.); hkoga@eco.sanken.osaka-u.ac.jp (H.K.)

**Keywords:** nanocellulose, cellulose nanofiber, transparent nanopaper, transparent conductive film

## Abstract

Nanopaper prepared from holocellulose pulp is one of the best substrates for flexible electronics because of its high thermal resistance and high clear transparency. However, the clearness of nanopaper decreases with increasing concentration of the starting cellulose nanofiber dispersion—with the use of a 2.2 wt % dispersion, for example—resulting in translucent nanopaper with a high haze of 44%. To overcome this problem, we show that the dilution of this high-concentration dispersion with water followed by sonication for 10 s reduces the haze to less than 10% while maintaining the high thermal resistance of the nanopaper. Furthermore, the combination of water dilution and a short sonication treatment improves the clearness of the nanopaper, which would translate into cost savings for the transportation and storage of this highly concentrated cellulose nanofiber dispersion. Finally, we demonstrate the improvement of the electrical conductivity of clear transparent nanopaper prepared from an initially high-concentration dispersion by dropping and heating silver nanowire ink on the nanopaper. These achievements will pave the way toward the realization of the mass production of nanofiber-based flexible devices.

## 1. Introduction

In our previous work, 3–15-nm-wide cellulose nanofibers were prepared by mechanical nanofibrillation from alkali-treated holocellulose pulps or 2,2,6,6-tetramethylpiperidine-1-oxyl (TEMPO)-oxidized cellulose pulps [1,2]. Aqueous dispersions of these cellulose nanofibers were dried to prepare films with high optical transparency and low haze [3,4] called “transparent nanopaper”. The high optical transparency originated from the nanofibers being so densely packed that light scattering inside the film or at the surface was prevented by the absence of cavities [4]. The nanopaper exhibited a low coefficient of thermal expansion similar to that of glass, higher thermal durability and a higher dielectric constant than those of most plastics, and excellent electrical insulation properties [4,5,6,7]. Moreover, the nanopaper remained lightweight with a high foldability similar to the properties of conventional paper. Given these advantages, flexible electronics based on cellulose nanopaper have been developed, including transparent electrodes, organic solar cells, transistors, antenna, and memory devices [6,7,8,9,10,11,12,13,14,15,16,17,18]. Transparent cellulose nanopaper is thus considered a promising substrate for future flexible devices.

To realize nanopaper-based flexible electronic devices, the thermal and moisture resistance, as well as the dielectric constants of clear transparent nanopaper have been improved, and lower-haze paper has been prepared [6,19,20,21]. However, the ability to fabricate clear transparent nanopaper from a low-concentration cellulose nanofiber dispersion remains an outstanding issue. Currently, the concentration of the starting dispersion is generally less than 0.5 wt % [3,4,5,11,13], which results in high transportation and storage costs for the dispersion, as well as high energy consumption during processing. To reduce these costs and the amount of energy consumed, the fabrication of clear transparent nanopaper should start from a high-concentration dispersion, which would enable the realization of nanopaper-based flexible devices.

In this study, we fabricated transparent nanopaper with low haze and high thermal resistance from a highly concentrated dispersion. We first evaluated the dependence of the nanopaper haze on the dispersion concentration using alkali-treated holocellulose nanofibers and TEMPO-oxidized nanofibers. Next, we developed a procedure to prepare low-haze transparent alkali-treated holocellulose nanopaper from high-concentration dispersions and revealed a dependence of the haze on the dispersion concentration. Finally, we evaluated the crystallinity and thermal resistance of the nanopapers and applied them in flexible transparent conductive films.

## 2. Materials and Methods

### 2.1. Cellulose Pulp

TEMPO-oxidized cellulose pulp was prepared using the steps described in a previous study [19]. Never-dried softwood dissolving sulfite pulp (dry weight 20 g) was suspended in distilled water (1500 mL) containing TEMPO (0.16 g) and NaBr (1.0 g). A NaClO solution (4 wt %) containing 0.4, 0.6, or 1.0 mmol/g cellulose (12.4, 18.48, or 30.8 mL) was then added to the cellulose slurry at room temperature under continuous stirring. Holocellulose pulp was also prepared using the steps described in a previous study [19,22]. First, 60 g of Japanese cedar (Cryptomeria japonica) wood chips were dewaxed in a mixture of acetone/water (2700 mL/300 mL) at room temperature overnight with gentle stirring. The chips were then delignified in an acetic anhydride/hydrogen peroxide mixture (500 mL/500 mL) at 90 °C for 2 h. Next, the holocellulose pulp was treated with 5 wt % potassium hydroxide at 20 °C for 2 h before being washed thoroughly with distilled water. The carboxylate content of the pulps was determined by the electric conductivity titration method [23].

### 2.2. Nanofibrillation

The disintegration of the cellulose pulps into nanofibers was performed using a water-jet nanofibrillation system [5,19]. First, 2000 g of the pulp slurry (pulp content: 0.27 wt %) was homogenized using a high-pressure water jet system (Star Burst, HJP-25008, Sugino Machine Co., Ltd., Toyama, Japan) equipped with a ball-collision chamber. The injected slurry was repeatedly passed through a small nozzle with a 0.15-mm diameter under a high pressure of 245 MPa. After 50 passes through this nozzle, a 0.20 wt % cellulose nanofiber water dispersion was obtained.

### 2.3. Conditioning of Cellulose Nanofiber Dispersion

The concentration of the cellulose nanofiber dispersion was adjusted by condensation using a rotary evaporator (EYLA SB1200, Tokyo Rikakikai Corp., Tokyo, Japan) and/or by water dilution. The 20-mL dispersion was sonicated for 5–30 s using an ultrasonic homogenizer (US-300E, Nissei Corp., Tokyo, Japan) with a 26-mm probe tip diameter at 19.5 kHz and 300-W output power. After the dispersion was conditioned by concentration adjustments and/or sonication, it was degassed using a centrifugal mixer (ARV-310, Thinky Corp., Tokyo, Japan) at 1400 rpm for 3 min under vacuum and then at 1800 rpm for 7 min under ambient pressure.

### 2.4. Nanopaper

The dispersion was then cast evenly on an acrylic plate with an applicator. Cellulose nanopapers with thicknesses of 13 ± 2 μm were obtained after subsequent oven-drying (DVS402, Yamato, Tokyo, Japan) at 55 °C under R.H. 25% overnight. The thickness of nanopaper was measured by a micrometer.

### 2.5. Characterization

The haze of the cellulose nanopaper was measured using a haze meter (HZ-V3, Suga Test Instruments Co., Ltd., Tokyo, Japan). X-ray diffraction patterns were recorded using a Rigaku MiniFlex600 (Tokyo, Japan) with Cu Kα radiation and a scanning angle (2θ) range of 5–40° at the 40-kV voltage and 15-mA current. The crystallinity index of cellulose I was calculated from the (200) reflection (2θ = ~22.6°), as previously described [20]. The thermal durability of the nanopaper was evaluated using the 5% weight-loss point. Thermogravimetric analysis (TGA Q50N2, TA Instruments, New Castle, DE, USA) was performed under a nitrogen atmosphere (60 mL/min) at a heating rate of 10 °C/min. The surface roughness of the nanopaper was determined using an atomic force microscope (AFM, Nanocute, SII Nano Technology Inc., Chiba, Japan) in the dynamic force microscope (DFM) mode (measurement range: 20 μm × 20 μm).

### 2.6. Silver Nanowire and Transparent Conductive Lines

Silver nanowires (50–100 nm in diameter and 5–10 μm in length) were synthesized via the reduction of silver nitrate in the presence of poly(vinylpyrrolidone) (PVP) in ethylene glycol [13,24]. The silver nanowires were dispersed in ethanol to form printable inks. The silver nanowire/ethanol suspension was dropped onto the transparent nanopaper and then air-dried for 3–5 min. The air-dried silver nanowires on the nanopapers were heated at 150 °C for 30 min in the air [13,24].

## 3. Results and Discussion

**Figure 1:**
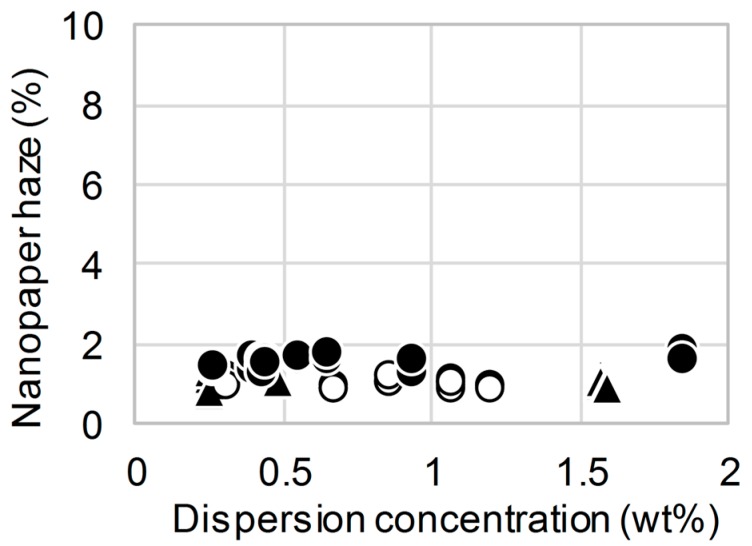
Effect of concentration of starting cellulose nanofiber dispersion on haze of 15-μm-thick TEMPO-oxidized nanopaper. Carboxyl content: (●) 0.4 mmol/g; (○) 0.6 mmol/g; (▲) 1.0 mmol/g.

Nanofibers (3–15-nm wide) were prepared by gentle mechanical nanofibrillation from the TEMPO-oxidized cellulose pulps [1,19]. The 0.1 wt % TEMPO-oxidized nanofiber dispersion (carboxyl content of 1.5 mmol/g) was dried to produce clearly transparent nanopaper, denoted TEMPO-oxidized nanopaper, with low haze (less than 5%) [3,19,21]. To investigate the dependence of the nanopaper haze on the concentration of the dispersion, TEMPO-oxidized nanopapers with thicknesses of 13 ± 2 μm were prepared from 0.2–1.8 wt % dispersions (carboxyl contents of 0.4–1.0 mmol/g). For the TEMPO-oxidized nanofibers containing 1.0 mmol/g carboxyl, all the dispersions (concentrations of 0.2–1.8 wt %) resulted in the production of transparent nanopaper with low haze values of less than 1.9% (Figure 1▲). Because of the electric double-layer repulsion, the TEMPO-oxidized nanofibers were homogeneously distributed in the water dispersions without the formation of inhomogeneous aggregations [25]. The cellulose nanofibers were so densely packed that there were no cavities, which generally cause light scattering either inside the film or at the surface [4,5]. Even for the carboxyl contents of 0.6 and 0.4 mmol/g, the repulsion of the cellulose nanofibers was retained. Therefore, the TEMPO-oxidized nanopapers maintained their low haze values of less than 1.9% for all the concentrations (Figure 1●,○), indicating that the use of these nanofibers would result in reduced transportation and storage costs of the dispersion as highly concentrated TEMPO-oxidized nanofiber dispersions could be used to prepare low-haze transparent nanopaper.

**Figure 2:**
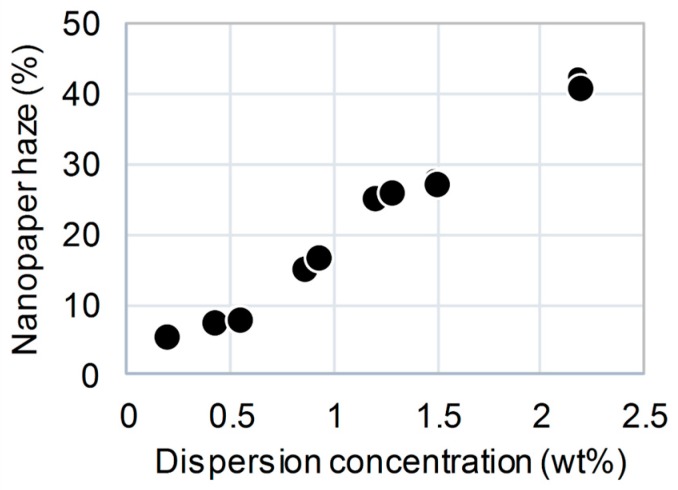
Effect of starting concentration of cellulose nanofiber dispersion on the haze of 15-μm-thick alkali-treated holocellulose nanopaper. Carboxyl content: 0.15 mmol/g.

Nanofibers (15-nm wide) were also prepared from the alkali-treated holocellulose pulps using mechanical nanofibrillation [2]. In the current process, less than 0.5 wt % alkali-treated holocellulose nanofiber dispersion is dried to produce clearly transparent nanopaper, denoted alkali-treated holocellulose nanopaper, with high thermal resistance and low haze (less than 10%) [5,19,20]. To investigate the concentration dependence of the resulting haze value, alkali-treated holocellulose nanopapers with thicknesses of 13 ± 2 μm were prepared from 0.2–2.2 wt % dispersions (Figure 2). Similar to the results reported in previous studies, low haze values of approximately 10% were achieved using the nanofiber dispersions with concentrations of less than 0.5 wt %. However, a drastic increase in the haze was observed when the 0.86 wt % nanofiber dispersion was used, and finally, the use of the high-concentration 2.2 wt % dispersion yielded a high haze of 41.0%. The carboxyl content of all the alkali-treated holocellulose pulps was 0.15 mmol/g. Therefore, the alkali-treated holocellulose nanofibers were homogeneously distributed in the low-concentration dispersions but tended to aggregate inhomogeneously in the high-concentration dispersions. As the cellulose nanofibers were not so densely packed, the presence of cavities between the nanofibers resulted in light scattering due to the refractive index difference between air and cellulose, leading to the formation of hazy transparent nanopaper.

**Figure 3:**
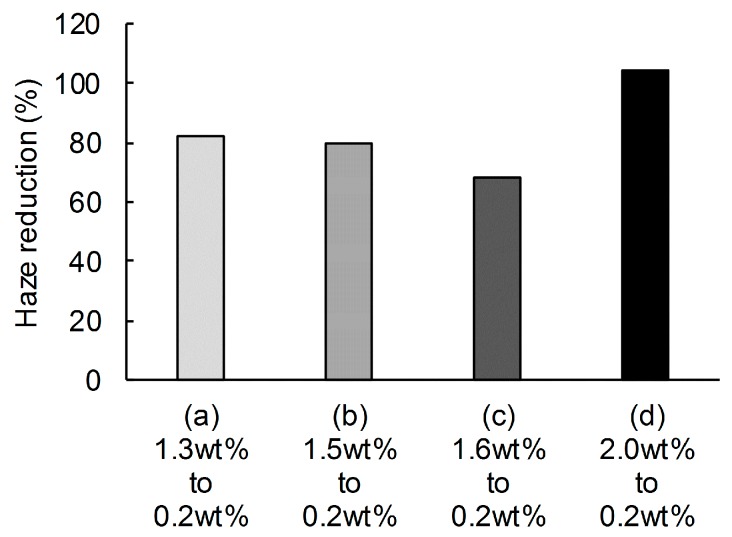
Reduction of the haze of holocellulose nanopaper prepared using 0.2 wt % cellulose nanofiber dispersions obtained by diluting dispersions of various initial concentrations. The dispersions with initial concentrations of (**a**) 1.3 wt % and (**c**) 1.6 wt % were prepared from holocellulose pulps without alkali treatments. The dispersions with initial concentrations of (**b**) 1.5 wt % and (**d**) 2.0 wt % were prepared from alkali-treated holocellulose pulps.

**Figure 4:**
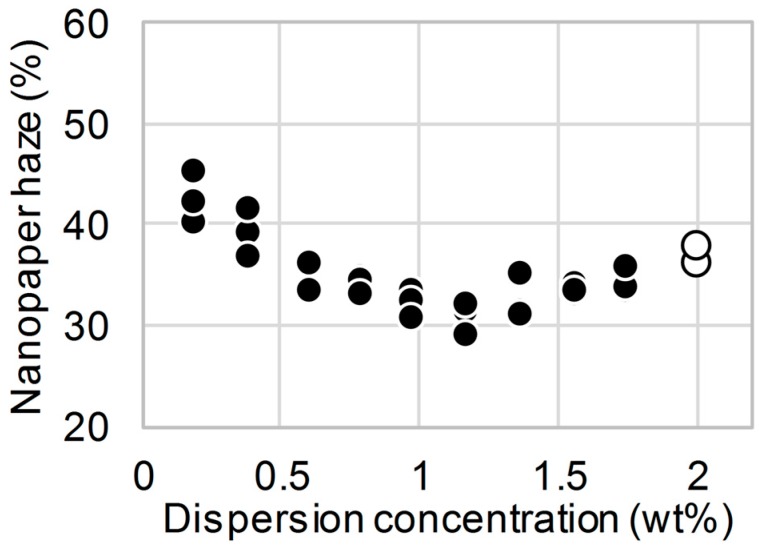
Effect of dispersion concentration on the haze of alkali-treated holocellulose nanopaper prepared using the original 2.0 wt % dispersion (open circle) and diluted 0.2–1.76 wt % dispersions (filled circles).

Inhomogeneous aggregations in cellulose nanofiber dispersions from holocellulose pulp without alkali-treatment are known to disintegrate upon water dilution [22]. When the concentrations of the dispersions with different initial concentrations of 1.3–1.6 wt % were diluted to 0.2 wt % and then subjected to oven drying, the nanopaper haze was reduced by 20–30%, regardless of alkali-treatment, compared with the values of nanopapers prepared without dilution (Figure 3a–c). However, as previously reported by Isobe et al., these nanopapers were still hazy translucent even after the water dilution [22], because the high-concentration dispersions without dilutions produced translucent nanopaper with haze values of approximately 30%. Moreover, when the concentration of the dispersion was diluted from 2.0 to 0.2 wt %, the haze of the nanopaper slightly increased rather than decreasing (Figure 3d). Moreover, even when the high-concentration 2.0 wt % dispersion was diluted to concentrations of 0.2–1.8 wt %, the diluted dispersions produced nanopapers with roughly constant high-haze values (Figure 4). These results suggest that the water dilution of high-concentration nanofiber dispersions does not lead to the disintegration of the inhomogeneous aggregations and cannot produce clearly transparent nanopaper with haze values of less than 10%.

**Figure 5:**
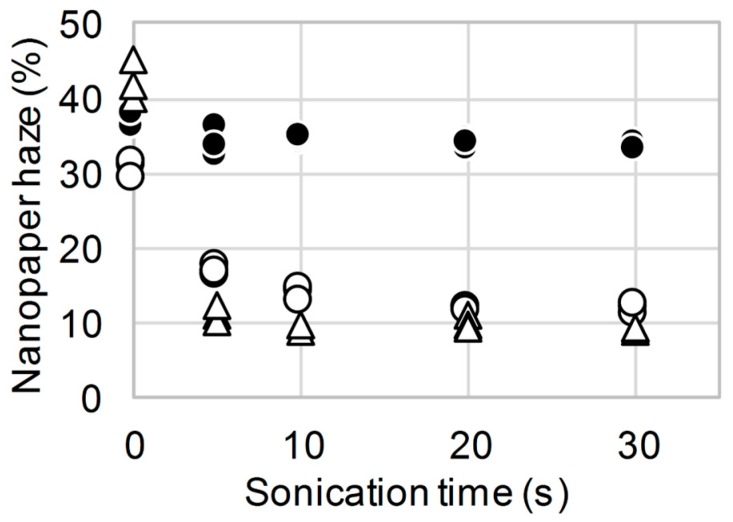
Effect of sonication time on the haze of alkali-treated holocellulose nanopaper prepared from dispersions with various concentrations: (●) 2.0 wt % dispersion without dilution, (○) 1.1 wt % dispersion diluted from 2.0 wt %, (△) 0.2 wt % dispersion diluted from 2.0 wt %.

Sonication is also often applied to disintegrate pulp fibers into nanofibers in water dispersions [3,21,26,27]. However, when the 2.0 wt % nanofiber dispersion was sonicated for 30 s, the obtained nanopaper maintained a high haze of approximately 35% because the high viscosity of the 2.0 wt % nanofiber dispersion resulted in absorption of the vibrations (Figure 5●). Therefore, we reduced the high viscosity of the dispersion using water to allow efficient propagation of the vibration through the dispersion, and the dispersion was then subjected to sonication. When the 1.1 and 0.2 wt % nanofiber dispersions diluted from the 2.0 wt % dispersion were sonicated, sonication for only 10 s resulted in the disintegration of the inhomogeneous aggregations in the dispersions and reduced the haze of the resulting transparent nanopaper to less than 10% (Figure 5○,△). In addition, the surface roughness (Rq) of the resulting nanopaper prepared from the diluted dispersion (2.0 to 0.2 wt %) and the diluted and sonicated dispersion was 43.5 and 30.2 nm, respectively. In a previous study, sonication was performed for 4–45 min because it was applied in a mechanical nanofibrillation process to produce cellulose nanofibers from microsized pulp fibers [3,21,25,26]. In contrast, the purpose of the sonication process in this study was to disintegrate the inhomogeneous aggregation between nanofibers in the dispersion; therefore, the use of a short sonication period of 10–30 s was sufficient.

**Figure 6:**
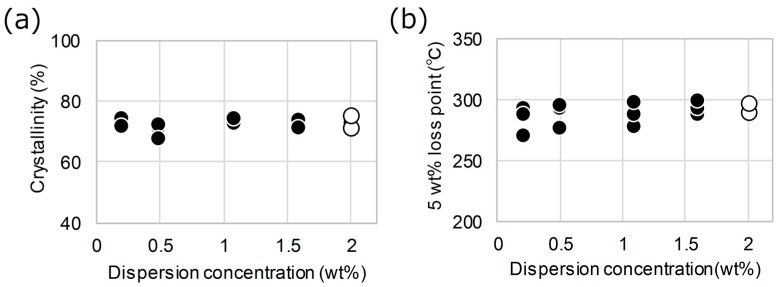
(**a**) Crystallinity and (**b**) heat resistance under nitrogen of transparent alkali-treated holocellulose nanopaper prepared using the original 2.0 wt % dispersion (open circle) and diluted 0.2–1.76 wt % dispersions (filled circles).

Such a short sonication time should only cause the dissolution of nanofiber aggregations without resulting in mechanical damage to the cellulose nanofibers. To confirm this, the 2.0 wt % dispersion was diluted to concentrations of 0.2–1.6 wt % and sonicated for 20 s, and then, transparent nanopapers were prepared by drying the dispersions. The resulting nanopapers exhibited almost the same high crystallinity and high heat resistance regardless of the conditions used (Figure 6a,b). Therefore, the dilution and sonication of the high-concentration dispersion improved the haze of the nanopaper while enabling its high crystallinity and heat resistance to be retained.

**Figure 7:**
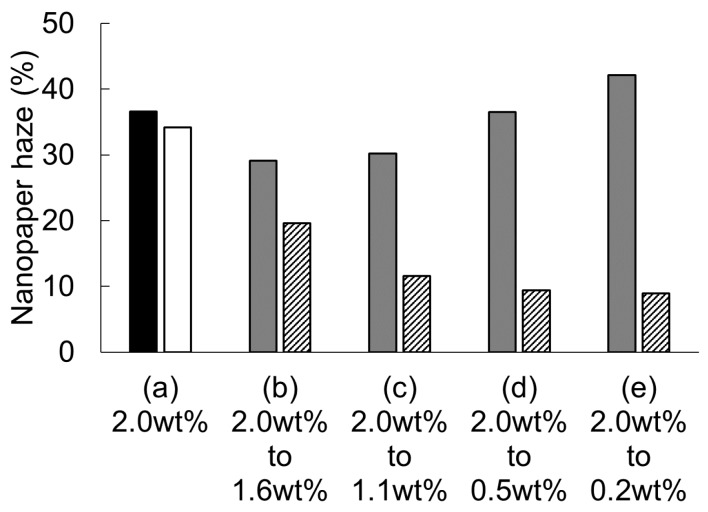
The haze of alkali-treated holocellulose nanopaper as a function of nanofiber dispersion conditions. (Black bar: Dispersion dried without any conditioning, White bar: Dispersion dried after 10-s sonication without dilution, Gray bar: Dispersions diluted and dried without sonication, Border bar: Dispersions diluted and dried after 10-s sonication).

Based on these results, we can summarize the procedure required to prepare low-haze transparent nanopaper from high-concentration dispersions. The drying of the 2.0 wt % cellulose nanofiber dispersion from the alkali-treated holocellulose pulps produced translucent nanopaper with a high haze of 36.6% (Figure 7a black bar). Because of the high viscosity of the 2.0 wt % dispersion, sonication for 10 s did not improve the nanopaper haze (34.2%, Figure 7a white bar). Dilution was also not very effective in improving the nanopaper haze (Figure 7b–e gray bars). For example, when the dispersion was diluted from 2.0 to 0.2 wt %, the haze of the nanopaper increased from 36.6% to 42.1%. However, the combination of dilution and sonication of the high-concentration dispersion drastically improved the nanopaper haze (Figure 7b–e border bars). When the dispersion was diluted from 2.0 to 0.5 wt % (or 0.2 wt %) and then sonicated for 10 s, the haze of the nanopaper decreased from 36.6% to 9.6% (or 9.3%); these haze values are equivalent to those reported in previous studies for nanopaper prepared from low-concentration (less than 0.5 wt %) nanofiber dispersions.

**Figure 8:**
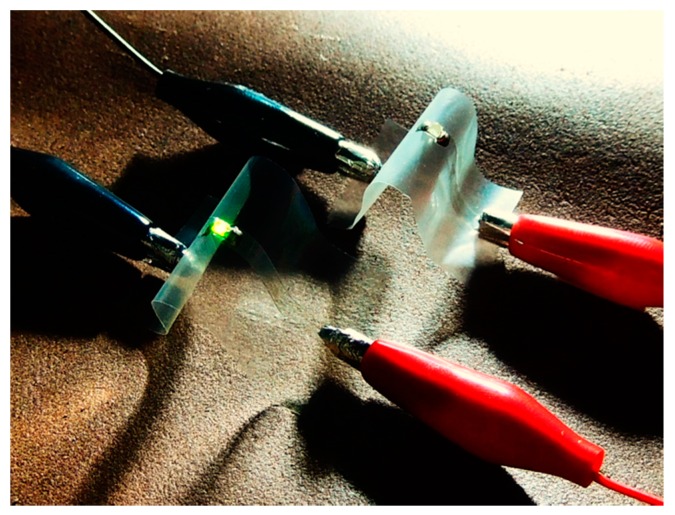
Illumination experiment using LED lights on conductive and transparent alkali-treated holocellulose nanopapers with silver nanowire deposits. The LED light on the low-conductive and translucent nanopaper prepared from the high-concentration dispersion without any conditioning was not illuminated (right). However, the LED light was successfully illuminated for the highly conductive and transparent nanopaper prepared from the diluted and sonicated high-concentration dispersion (left).

Although the ability to produce low-haze transparent nanopaper from high-concentration TEMPO-oxidized nanofiber dispersions (Figure 1) will help reduce the costs related to the transportation and storage of such dispersions, the heat sensitivity of TEMPO nanopaper still restricts its application as a transparent substrate for electronic devices [19]. In contrast, the high thermal resistance of alkali-treated holocellulose nanopapers has enabled their use as substrates for various electronic devices such as organic thin-film transistors, solar cells, and conductive lines [10,13,19,27]. As a specific example of our achievement for electronic applications, transparent and conductive nanopaper was fabricated by depositing silver nanowires on transparent nanopaper. Transparent conductive films are generally produced by dropping silver nanowire suspensions onto transparent substrates followed by heating at 150–200 °C in air. Such high-temperature heating could not be applied for the TEMPO nanopaper, because the nanopaper turned yellow or brown, but it could be used for the alkali-treated holocellulose nanopaper because the nanopaper maintained its colorless transparency. The transparency of silver-nanowire transparent conductive films depends on the transparency of the substrate and the silver nanowire content, and their electrical conductivity depends on the silver nanowire content [24]. Therefore, when the high-haze nanopaper prepared by drying the dispersion without any conditioning was used as a transparent substrate, only a small amount of deposited silver nanowires were used to enhance the transparency of the transparent and conductive nanopaper. As a result, the electrical conductivity of the transparent and conductive nanopaper was not sufficient to illuminate a light-emitting diode (LED) light (Figure 8 right). In contrast, when the low-haze nanopaper obtained by drying the diluted and sonicated dispersion was used as the transparent substrate, the high silver nanowire content enhanced the electrical conductivity of the transparent and conductive paper while enabling its high transparency to be maintained. Thus, a LED light mounted on the paper was successfully illuminated (Figure 8 left). These preliminary achievements suggest that the dilution and sonication of high-concentration nanofiber dispersions would contribute not only to reducing transportation and storage costs but also to expanding device applications.

## 4. Conclusions

Clearly transparent nanopapers have been fabricated using low-concentration (less than 0.5 wt %) cellulose nanofiber dispersions prepared from alkali-treated holocellulose pulps. The high cost of clearly transparent nanopaper resulting from the high transportation and storage costs of low-concentration dispersions is one of the obstacles currently preventing the mass production of flexible paper electronics. Therefore, procedures to prepare clearly transparent nanopaper from highly concentrated nanofiber dispersions are needed. However, increasing the concentration of nanofiber dispersions results in the generation of inhomogeneous aggregations of cellulose nanofibers in the dispersion, thereby decreasing the clearness of the transparent nanopaper. The inhomogeneous aggregations were not disintegrated by dilution of the high-concentration dispersions. In addition, although sonication often causes disintegration of aggregations of cellulose nanofibers, the inhomogeneous aggregations in the high-concentration dispersion in this study could not be disintegrated by sonication because of its high viscosity. However, after dilution of the high-concentration dispersion, the use of only 10–30-s sonication resulted in the production of clearly transparent nanopaper with a haze of less than 10%. Because these procedures allowed the high crystallinity of the nanopaper to be maintained, the high heat resistance of the clearly transparent nanopaper was also retained. Moreover, we prepared highly electrically conductive lines using the clearly transparent and high-heat-resistant nanopaper without sacrificing the transparency of the nanopaper. These achievements will help reduce the manufacturing costs associated with transparent nanopaper and encourage the widespread use of future flexible electronics based on nanopaper substrates.

## References

[1] Saito T., Nishiyama Y., Putaux J.L., Vignon M., Isogai A. (2006). Homogeneous suspensions of individualized microfibrils from TEMPO-catalyzed oxidation of native cellulose. Biomacromolecules.

[2] Abe K., Iwamoto S., Yano H. (2007). Obtaining cellulose nanofibers with a uniform width of 15 nm from wood. Biomacromolecules.

[3] Fukuzumi H., Saito T., Iwata T., Kumamoto Y., Isogai A. (2009). Transparent and high gas barrier films of cellulose nanofibers prepared by TEMPO-mediated oxidation. Biomacromolecules.

[4] Nogi M., Iwamoto S., Nakagaito A.N., Yano H. (2009). Optically transparent nanofiber paper. Adv. Mater..

[5] Nogi M., Kim C., Sugahara T., Inui T., Takahashi T., Suganuma K. (2013). High thermal stability of optical transparency in cellulose nanofiber paper. Appl. Phys. Lett..

[6] Inui T., Koga H., Nogi M., Komoda N., Suganuma K. (2015). A miniaturized flexible antenna printed on a high dielectric constant nanopaper composite. Adv. Mater..

[7] Celano U., Nagashima K., Koga H., Nogi M., Zhuge F., Meng G., He Y., De Boeck J., Jurczak M., Vandervorst W. (2016). All-nanocellulose nonvolatile resistive memory. NPG Asia Mater..

[8] Zhu H., Fang Z., Preston C., Lia Y., Hu L. (2014). Transparent paper: Fabrications, properties, and device applications. Energy Environ. Sci..

[9] Fang Z., Zhu H., Preston C., Hu L. (2014). Development, application and commercialization of transparent paper. Transl. Mater. Res..

[10] Fujisaki Y., Koga H., Nakajima Y., Nakata M., Tsuji H., Yamamoto T., Kurita T., Nogi M., Shimidzu N. (2014). Transparent nanopaper-based flexible organic thin-film transistor array. Adv. Funct. Mater..

[11] Koga H., Nogi M., Komoda N., Nge T.T., Sugahara T., Suganuma K. (2014). Uniformly connected conductive networks on cellulose nanofiber paper for transparent paper electronics. NPG Asia Mater..

[12] Nagashima K., Koga H., Celano U., Zhuge F., Kanai M., Rahong S., Meng G., He Y., De Boeck J., Jurczak M. (2014). Cellulose nanofiber paper as an ultra flexible nonvolatile memory. Sci. Rep..

[13] Nogi M., Karakawa M., Komoda N., Yagyu H., Nge T.T. (2015). Transparent conductive nanofiber paper for foldable solar cells. Sci. Rep..

[14] Jung Y.H., Chang T.H., Zhang H., Yao C., Zheng Q., Yang V.W., Mi H., Kim M., Cho S.J., Park D.W. (2015). High-performance green flexible electronics based on biodegradable cellulose nanofibril paper. Nat. Commun..

[15] Kang W., Yan C., Foo C.Y., Lee P.S. (2015). Foldable electrochromics enabled by nanopaper transfer method. Adv. Funct. Mater..

[16] Hsieh M.C., Kim C., Nogi M., Suganuma K. (2013). Electrically conductive lines on cellulose nanopaper for flexible electrical devices. Nanoscale.

[17] Hoeng F., Denneulina A., Bras J. (2016). Use of nanocellulose in printed electronics: A review. Nanoscale.

[18] Chinga-Carrasco G., Tobjörk D., Österbacka R. (2012). Inkjet-printed silver-nanoparticles on nano-engineered cellulose films for electrically conducting structures and organic transistors—Concept and challenges. J. Nanopart. Res..

[19] Yagyu H., Saito T., Isogai A., Koga H., Nogi M. (2015). Chemical Modification of Cellulose Nanofibers for the Production of Highly Thermal Resistant and Optically Transparent Nanopaper for Paper Devices. ACS Appl. Mater. Interfaces.

[20] Yagyu H., Ifuku S., Nogi M. (2017). Acetylation of Optically Transparent Cellulose Nanopaper for High Thermal and Moisture Resistance in a Flexible Device Substrate. Flex. Print. Electron..

[21] Zhao M., Ansari F., Takeuchi M., Shimizu M., Saito T., Berglund L.A., Isogai A. (2018). Nematic structuring of transparent and multifunctional nanocellulose papers. Nanoscale Horiz..

[22] Isobe N., Kasuga T., Nogi M. (2018). Clear transparent cellulose nanopaper prepared from concentrated dispersion by high-humidity drying. RSC Adv..

[23] Saito T., Isogai A. (2004). TEMPO-Mediated Oxidation of Native Cellulose. The Effect of Oxidation Conditions on Chemical and Crystal Structures of the Water-Insoluble Fractions. Biomacromolecules.

[24] Tokuno T., Nogi M., Karakawa M., Jiu J., Nge T.T., Aso Y., Suganuma K. (2011). Fabrication of silver nanowire transparent electrodes at room temperature. Nano Res..

[25] Tanaka R., Saito T., Hänninen T., Ono Y., Hakalahti M., Tammelin T., Isogai A. (2016). Viscoelastic Properties of Core–Shell-Structured, Hemicellulose-Rich Nanofibrillated Cellulose in Dispersion and Wet-Film States. Biomacromolecules.

[26] Zhao H.P., Feng X.Q., Gao H. (2007). Ultrasonic technique for extracting nanofibers from nature materials. Appl. Phys. Lett..

[27] Nge T.T., Nogi M., Suganuma K. (2013). Electrical functionality of inkjet-printed silver nanoparticle conductive tracks on nanostructured paper compared with those on plastic substrates. J. Mater. Chem. C..

